# Supplementary material for: A Salmonella Microfluidic Chip Combining Non-Contact Eddy Heater and 3D Fan-Shaped Mixer with Recombinase Aided Amplification
Source: Biosensors (Basel). 2022 Sep 5;12(9):726. doi: 10.3390/bios12090726 (PMC9496460; doi:10.3390/bios12090726)
Supplement: Supplementary file 1 [file biosensors-12-00726-s001.zip › biosensors-1851670-supplementary.pdf]

Supporting Information

A *Salmonella* Microfluidic Chip Combining Non-Contact Eddy Heater and 3D Fan-Shaped Mixer with Recombinase Aided Amplification

Shangyi Wu[1,2], Hong Duan[2], Yingchao Zhang[2], Siyuan Wang[2], Lingyan Zheng[3], Gaozhe Cai[4], Jianhan Lin[2, *], Xiqing Yue[1,#]

[1] College of Food Science, Shenyang Agricultural University, Shenyang 110866, China

[2] Key Laboratory of Agricultural Information Acquisition Technology, Ministry of Agriculture and Rural Affairs, China Agricultural University, Beijing 100083, China

[3] Beijing Engineering and Technology Research Center of Food Additives, Beijing Technology & Business University, Beijing 100048, China

[4] State Key Laboratory of Transducer Technology, Shanghai Institute of Microsystem and Information Technology, Chinese Academy of Sciences, Shanghai 200050, China

*: corresponding author: Dr. Jianhan Lin, email: jianhan@cau.edu.cn

#:co-corresponding author: Dr. Xiqing Yue, email: yxqsyau@126.com

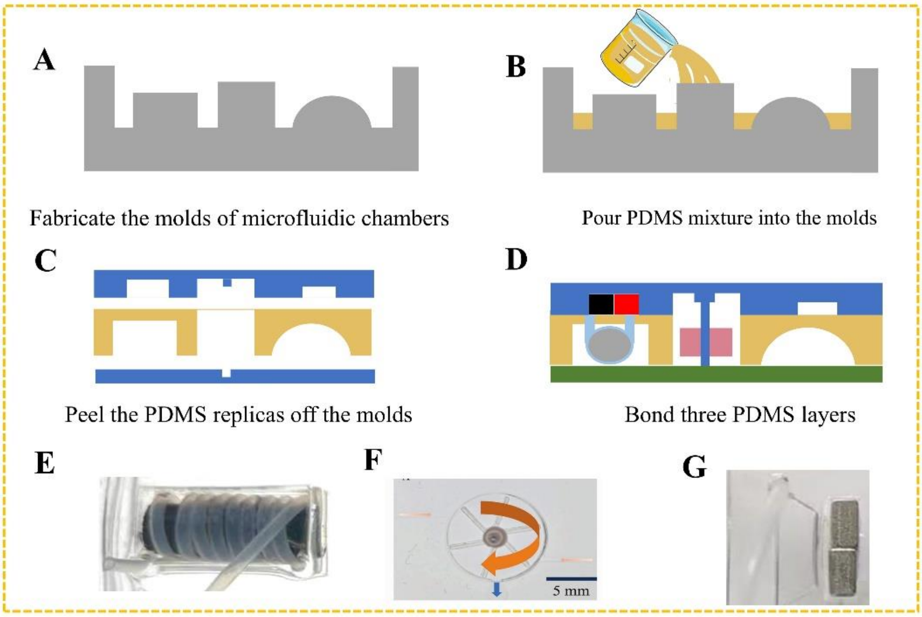

Figure S1. The diagram of the microfluidic chip fabrication. (A) The molds of the microfluidic chambers were 3D printed. (B) The mixture of PDMS prepolymer and curing agent was poured into the molds. (C) The PDMS replicas were peeled off the molds after curing for 2 h (in a 65°C oven). (D) The three layers were bonded together after the iron rod tightly wound by Teflon tubing and the fan-shaped mixer were placed inside the middle PDMS layer and the magnet was placed inside the top PDMS layer. (E) The photo of the iron rod tightly wound by Teflon tubing. (F) The photo of the fan-shaped mixer. (G) The photo of the magnet.

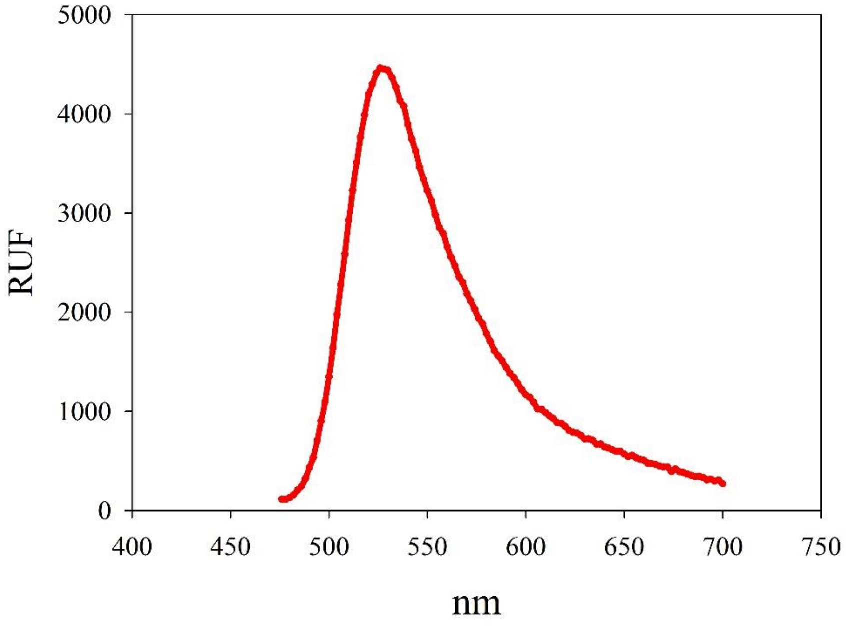

Figure S2. The fluorescence spectrum of the EvaGreen fluorescent dye from the fluorescence reader

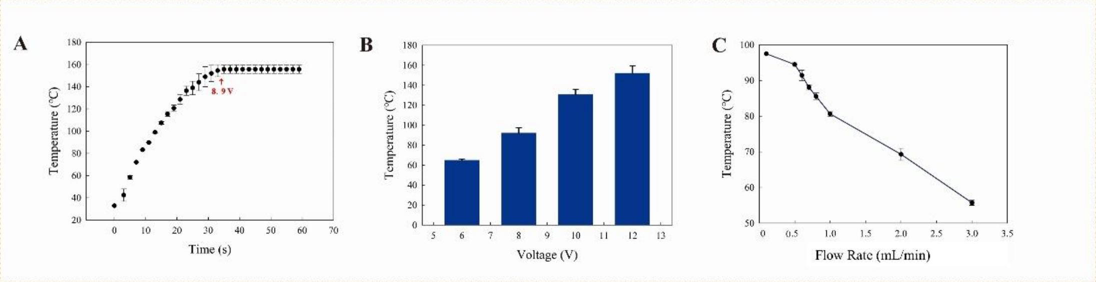

Figure S3. The performance of the eddy heater. (A)The temperature of the iron rod at different time after 12 V was applied on the heater (N=3). (B) The average temperature of the iron rod at 30 s (N=3). (C) The temperature of deionized water at the outlet for different flow rates(N=3).

To provide a stable temperature for bacterial lysis, different voltages were applied on the alternating electromagnetic field generator for different time. As shown in Figure S3A, the starting voltage of 12 V was applied on the alternating electromagnetic field generator and the temperature was monitored using the smartphone infrared thermal imager. After the temperature reached up to 150°C in 30 s, the voltage was reduced to 8.9 V to make the temperature be stable at 150°C. To optimize the voltage applied on the generator, different voltages ranging from 6 V to 12 V were used for heating. As shown in Figure S3B, the temperature increased with the voltage, and after trading off the convenience and portability (12 V power supplies were accessible and low-cost), the voltage of 12 V was used in this study. To optimize the flow rate, deionized water was injected into the tubing at different flow rates, and its temperature at the outlet was measured using the thermocouple probe. As shown in Fig. S3C, the temperature decreased with the flow rate. Since the temperatures at the flow rate of >0.5 mL/min was lower than 90°C and the bacterial cells might not be lysed sufficiently, the flow rate of 0.5 mL/min was used in this study.

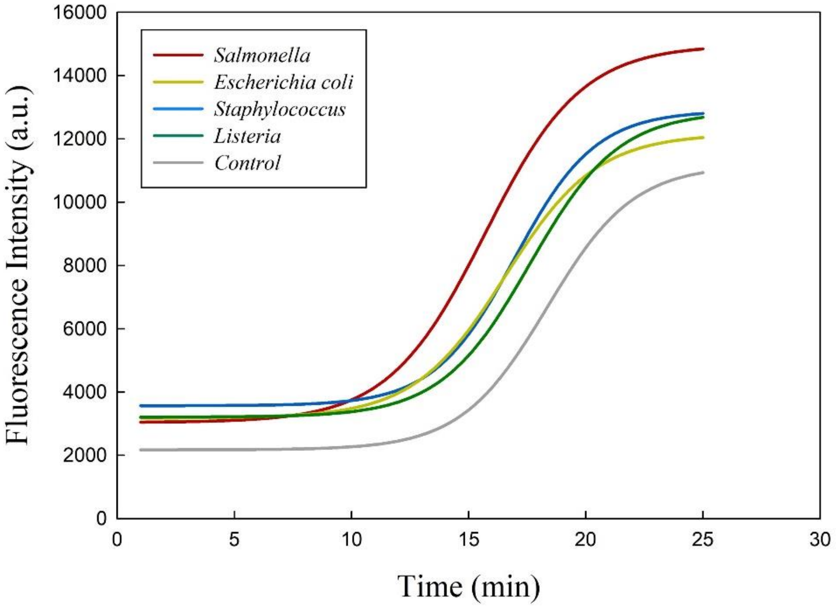

Figure S4. The original amplification curves measured using the fluorescent reader

Table S1 Comparison of reported bacterial detection methods combining microfluidics with RAA

Methods	Targets	Included steps	Time (min)	Detection limit (CFU/mL)	Ref
Centrifugal microfluidics	*Listeria spp.*	Amplification, detection	~60	4.9×10^3	[1]
Centrifugal microfluidics	*Pseudomonas aeruginosa*	Amplification, detection	~90	$\sim 10^3$	[2]
Centrifugal microfluidics	*Salmonella*	Amplification, detection	15-40	3.5×10^1	[3]

Table S2 The primer sets sequence

	Nucleotide sequence (5’-3’)
Forward primer	ATTGGCGATAGCCTGGCGGTGGGTTTTGTTGT
Reverse primer	TACCGGGCATACCATCCAGAGAAAATCGGGCCGC
Complete nucleotide sequence	ATTGGCGATAGCCTGGCGGTGGGTTTTGTTGTCTTCTCTATTGTCACCGTGGTCCAGTTTATCGTTA<br>TTACCAAAGGTTCAGAACGTGTCGCGGAAGTCGCGGCCCGATTTTCTCTGGATGGTATGCCCGGTA

References

1. Xiang, X.; Li, F.; Ye, Q.; Shang, Y.; Chen, M.; Zhang, J.; Zhou, B.; Suo, H.; Ding, Y.; Wu, Q. High-Throughput Microfluidic Strategy Based on RAA-CRISPR/Cas13a Dual Signal Amplification for Accurate Identification of Pathogenic Listeria. *Sensors and Actuators B: Chemical* **2022**, *358*, 131517, doi:10.1016/j.snb.2022.131517.
2. Chen, Y.; Mei, Y.; Zhao, X.; Jiang, X. Reagents-Loaded, Automated Assay That Integrates Recombinase-Aided Amplification and Cas12a Nucleic Acid Detection for a Point-of-Care Test. *Anal. Chem.* **2020**, *92*, 14846–14852, doi:10.1021/acs.analchem.0c03883.
3. Xiang, X.; Shang, Y.; Ye, Q.; Li, F.; Zhang, J.; Zhou, B.; Suo, H.; Chen, M.; Gu, Q.; Ding, Y.; et al. A *Salmonella* Serogroup Rapid Identification System for Food Safety Based on High Throughput Microfluidic Chip Combined with Recombinase Aided Amplification. *Sens. Actuators, B* **2022**, *357*, 131402, doi:10.1016/j.snb.2022.131402.
